# 

**Published:** 1888-09-15

**Authors:** 


### THE HOSPITAL

A Weekly Institutional Journal of
### Science, Medicine, Nursing, and Philanthropy.

### SPECIAL STUDENTS' NUMBER.

### CONTENTS.

**An "Unvarnished Tale"** - 381

**The Student's Bitter Cry** - 382

**Medical Schools and Medical Students.**—A Parent's Protest—What the Deans Say - 383

**The Discipline of Medical Schools** - 385

**The Plea of the Patient** - 387

**Notes and News** - 388

**Everybody's Page** - 390

**Annotations** - 391

**Hints for the Sick-room.** By M. M. Basil, M.A., M.B. 392

**An Ishmaelite.** By Leslie Keith - 393

**A Garden Party at the Cancer Hospital** - 394

**Malingering Under the Old Regime.**—By W. Curran, A.M.D. - 395

**Hospital Clothing and Bedding.—A Hypochondriacal Patient's Song. -Children's Corner** - 396

**EXTRA SUPPLEMENT.—THE NURSING MIRROR.**

En Passant - xciii

Original Articles.—A Sister's Holiday. Part I - xciv

Letters from a Probationer.—No III. The Hospital Chapel - xcv

Glasgow Royal Infirmary.—Opening of New Nurses' Home - xcv

Everybody's Opinion.—The Red Cross.—Zenana Medical College - xcvi

Lectures.—Vacancies—Notes and Queries.—Appointments - xcvi

